# Dissection of protein and RNA regions required for SPEN binding to XIST A-repeat RNA

**DOI:** 10.1261/rna.079713.123

**Published:** 2024-03

**Authors:** Aileen C. Button, Simone D. Hall, Ethan L. Ashley, Colleen A. McHugh

**Affiliations:** Department of Chemistry and Biochemistry, University of California San Diego, La Jolla, California 92093, USA

**Keywords:** RNA recognition motif, RNA–protein interactions, SPEN, SHARP, X chromosome silencing, XIST noncoding RNA

## Abstract

XIST noncoding RNA promotes the initiation of X chromosome silencing by recruiting the protein SPEN to one X chromosome in female mammals. The SPEN protein is also called SHARP (SMRT and HDAC-associated repressor protein) and MINT (Msx-2 interacting nuclear target) in humans. SPEN recruits N-CoR2 and HDAC3 to initiate histone deacetylation on the X chromosome, leading to the formation of repressive chromatin marks and silencing gene expression. We dissected the contributions of different RNA and protein regions to the formation of a human XIST–SPEN complex in vitro and identified novel sequence and structure determinants that may contribute to X chromosome silencing initiation. Binding of SPEN to XIST RNA requires RRM 4 of the protein, in contrast to the requirement of RRM 3 and RRM 4 for specific binding to SRA RNA. Measurements of SPEN binding to full-length, dimeric, trimeric, or other truncated versions of the A-repeat region revealed that high-affinity binding of XIST to SPEN in vitro requires a minimum of four A-repeat segments. SPEN binding to XIST A-repeat RNA changes the accessibility of the RNA at specific nucleotide sequences, as indicated by changes in RNA reactivity through chemical structure probing. Based on computational modeling, we found that inter-repeat duplexes formed by multiple A-repeats can present an unpaired adenosine in the context of a double-stranded region of RNA. The presence of this specific combination of sequence and structural motifs correlates with high-affinity SPEN binding in vitro. These data provide new information on the molecular basis of the XIST and SPEN interaction.

## INTRODUCTION

Mammals use an RNA-based silencing mechanism to equalize gene expression dosage in females with two X chromosomes (XX) compared to males with one X chromosome (XY). Silencing of all but one X chromosome in XX, XXX, or XXY individuals is termed X chromosome inactivation (XCI) and takes place during early embryonic development (Lyon 1962). This silencing process ensures that only a single allele for most X-linked genes will be transcribed in each somatic female cell. In humans, XCI occurs randomly, and either the maternal or paternal X chromosome becomes silenced (Moreira de Mello et al. 2010). The silenced X chromosome is condensed to form a Barr body (Barr and Bertram 1949) and this transcriptional silencing is maintained during clonal expansion through development.

Expression of the XIST noncoding RNA is essential for XCI in humans and other eutherian mammals (Penny et al. 1996; Sahakyan et al. 2018), while metatherian mammals rely on the Rsx noncoding RNA for a similar process (Grant et al. 2012). Human XIST is a 17-kilonucleotide RNA containing multiple regions of conserved repeat sequences with functions in silencing (Brockdorff et al. 1992; Brown et al. 1992). During initiation of XCI, XIST RNA directly binds and recruits SPEN to the inactive X chromosome (McHugh et al. 2015). Specifically, the A-repeat region of XIST is required for recruitment of SPEN and initiation of silencing (Wutz et al. 2002; Chu et al. 2015). In humans, the XIST A-repeat region consists of eight and a half A-repeat sequences with intervening U-rich linkers. This linear structure, consisting of alternating repeats and U-rich linker regions, is conserved among species. In other mammalian species such as chiropters and lagomorphs, the A-repeat region can contain as few as six full repeats. While the overall structure of each XIST A-repeat is conserved, nucleotide sequence variations occur across individual repeats even in the same species (Supplemental Fig. S1). The unusual sequence and structure of the A-repeat region of XIST RNA may be critical for SPEN binding and recruitment to the X chromosome. A single XIST A-repeat sequence can form a double hairpin while multiple repeats can form inter-repeat duplexes (Duszczyk et al. 2008, 2011). Higher order and more complex structures have been proposed to form through base complementarity with neighboring or distant repeats (Maenner et al. 2010; Fang et al. 2015). Structural analyses of the A-repeat region suggested that this region can fold into a compact RNA structure, independent of the remainder of the XIST RNA transcript (Lu et al. 2016; Liu et al. 2017).

SPEN is a human homolog of the *Drosophila* spen (split ends) protein. This protein is also called SHARP (SMRT and HDAC-associated repressor protein) and MINT (Msx-2 interacting nuclear target) in humans. SPEN is a large multidomain RNA-binding protein (RBP). The SPEN family proteins regulate gene expression in several developmental processes. The *Drosophila* spen protein was first identified as a positive regulator of the DER/Ras signaling pathway (Chen and Rebay 2000) and interacts with Hox to regulate segmental morphologies (Wiellette et al. 1999). SPEN can act as a transcriptional repressor (Ariyoshi and Schwabe 2003). SPEN contains several conserved protein domains that enable interactions with RNA coactivators and the nuclear hormone coreceptors N-CoR2 and histone deacetylases (HDACs) to silence transcription (Shi et al. 2001). SPEN is a predominantly nuclear protein, containing four RNA recognition motif (RRM) domains at the N-terminus and a SPOC domain at the C-terminus. The SPOC domain allows SPEN to achieve transcriptional repressor activity by recruiting regulatory proteins to the X chromosome (Dossin et al. 2020).

RNA binding of SPEN occurs through the four N-terminal RRM domains. The N-terminal region of the protein contains RRM 1 followed by a disordered linker region, RRM 2, a second linker region, RRM 3, and RRM 4. The SPEN_RRM 2–4_ construct has been crystallized and can bind tightly to the steroid receptor antigen (SRA) noncoding RNA (Shi et al. 2001; Arieti et al. 2014). The mechanism by which SPEN recognizes and binds XIST is still unknown. RRM domains can recognize and bind a wide range of nucleotide sequences (Afroz et al. 2015) making prediction of specific RNA–protein interactions from sequence information a challenge. For human XIST RNA, the number and identity of individual A-repeat sequences within the XIST A-repeat region that are required to achieve high-affinity binding to SPEN in vitro were not previously investigated systematically. Since the XIST–SPEN interaction is critical for X chromosome silencing, we performed truncation and binding studies of the RNA and protein in vitro to investigate the molecular basis for this RNA–protein interaction. We also examined the consequences of protein binding on XIST A-repeat RNA structure and identified a specific structural and sequence motif that was correlated with high affinity in vitro SPEN binding to XIST A-repeat sequences.

## RESULTS

### XIST_A-full_ and SRA RNA bind SPEN with similar affinity

**FIGURE 1.**
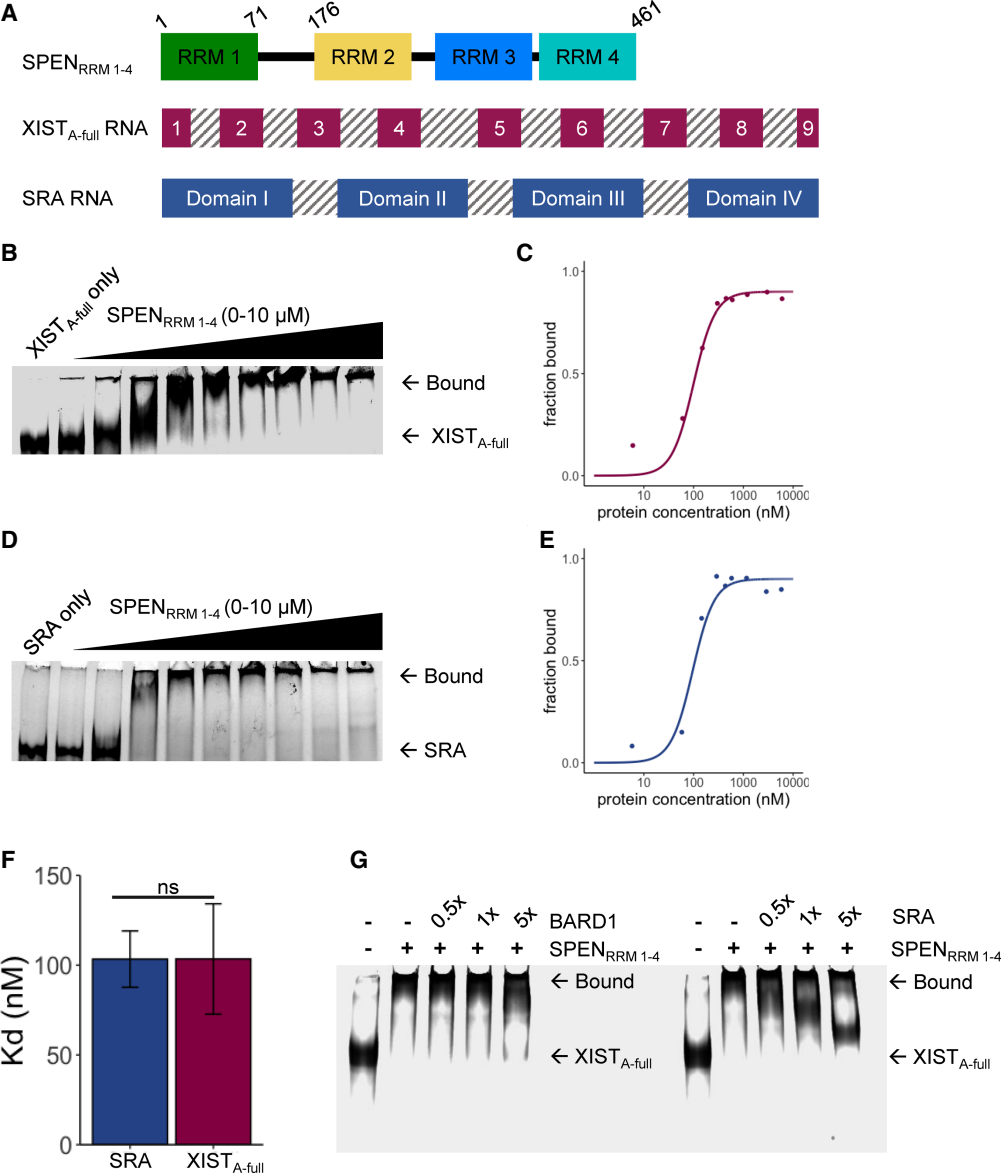
XIST_A-full_ and SRA RNA bind SPEN_RRM 1–4_ with similar affinity. (*A*) Constructs of SPEN_RRM 1–4_, the XIST A-repeat region (XIST_A-full_), and SRA RNA used for binding studies. (*B*) EMSA gel-shift assay of XIST_A-full_ and SPEN_RRM 1–4_ interaction. (*C*) Quantification of bound fraction of XIST_A-full_ and SPEN_RRM 1–4_ with increasing protein concentration, *n* = 6. Data are the mean ± SEM. (*D*) EMSA gel-shift assay of SRA and SPEN_RRM 1–4_. (*E*) Quantification of bound fractions of SRA and SPEN_RRM 1–4_, *n* = 6. Data are the mean ± SEM. (*F*) Binding affinity of XIST_A-full_ and SRA RNA for SPEN_RRM 1–4_ protein construct, *n* = 6. (n.s.) Not significant, *P* > 0.05. Data are the mean ± SEM. (*G*) Competitive gel-shift assay between XIST_A-full_ (labeled) and SRA or BARD1 (unlabeled competitor) for binding to SPEN_RRM 1–4_.

To investigate the binding regions required for formation of stable SPEN and human XIST RNA complexes in vitro, we cloned the N-terminal region of human SPEN to create the SPEN_RRM 1–4_ protein construct, along with the human XIST A-repeat RNA region and SRA RNA transcript (Fig. 1A). Previous work suggested that the A-repeat region folds independently and may form complex inter-repeat interactions. We performed in vitro binding studies using a multiplexed version of the electrophoretic mobility shift assay (EMSA) where trace amounts of fluorescently labeled RNA were incubated with increasing concentrations of SPEN protein. The resulting RNA–protein complexes were separated by native polyacrylamide gel electrophoresis (Fig. 1B). BSA binding to SRA RNA was used as a negative control for the in vitro RNA–protein binding experiments (Supplemental Fig. S2). We quantified the bound fraction of RNA and calculated the binding affinity of SPEN_RRM 1–4_ for XIST_A-full_. The equilibrium dissociation constant (*K*_*d*_) of the SPEN_RRM 1–4_ and XIST_A-full_ RNA–protein interaction was 100 ± 30 nM (Fig. 1C; Table 1). The same experiments were also performed for SRA RNA binding to SPEN_RRM 1–4_, and the *K*_*d*_ for this interaction was 100 ± 20 nM (Fig. 1D,E). SRA RNA and XIST_A-full_ RNA each bound to SPEN_RRM 1–4_ with similar affinity in EMSA studies (Fig. 1F). A size-matched fragment from an unrelated messenger RNA, BARD1, was used as a negative control for SPEN binding (Supplemental Fig. S2). Since SRA and XIST RNA were predicted to bind to SPEN at the same site (Monfort et al. 2015; Carter et al. 2020), we performed a competition assay between XIST and SRA for SPEN binding. Labeled XIST_A-full_ RNA was mixed with SPEN_RRM 1–4_, then increasing quantities of unlabeled SRA or BARD1 negative control RNA were added to the samples to determine whether SRA or BARD1 binding could outcompete XIST binding. Addition of SRA RNA to the complexes could displace the binding of XIST_A-full_ RNA to SPEN_RRM 1–4_ protein, indicating that XIST_A-full_ and SRA binding to SPEN were mutually exclusive (Fig. 1G). The control experiment performed with BARD1 negative control RNA showed that BARD1 could not compete with XIST_A-full_ for binding to SPEN.

**TABLE 1.** *K*_d_ of XIST_A-full_, SRA, and BARD1 binding to SPEN protein constructs

Protein construct	RNA	*K*_*d*_ (nM)	Hill's coefficient
SPEN_RRM 1–4_	XIST_A-full_	100 ± 30	1
SPEN_RRM 2–4_	XIST_A-full_	120 ± 30	1
SPEN_RRM 2–4 mut 3_	XIST_A-full_	230 ± 40	1
SPEN_RRM 2–3_	XIST_A-full_	1050 ± 110	2
SPEN_RRM 1–4_	SRA	100 ± 20	2
SPEN_RRM 2–4_	SRA	300 ± 30	2
SPEN_RRM 2–4 mut 3_	SRA	950 ± 100	3
SPEN_RRM 2–3_	SRA	5570 ± 700	1
SPEN_RRM 1–4_	BARD1	210 ± 60	2
SPEN_RRM 2–4_	BARD1	550 ± 30	10
SPEN_RRM 2–4 mut 3_	BARD1	2300 ± 500	2
SPEN_RRM 2–3_	BARD1	10,000 ± 0	2

### SPEN_RRM 1–4_ increases SRA RNA binding compared to SPEN_RRM 2–4_ construct

**FIGURE 2.**
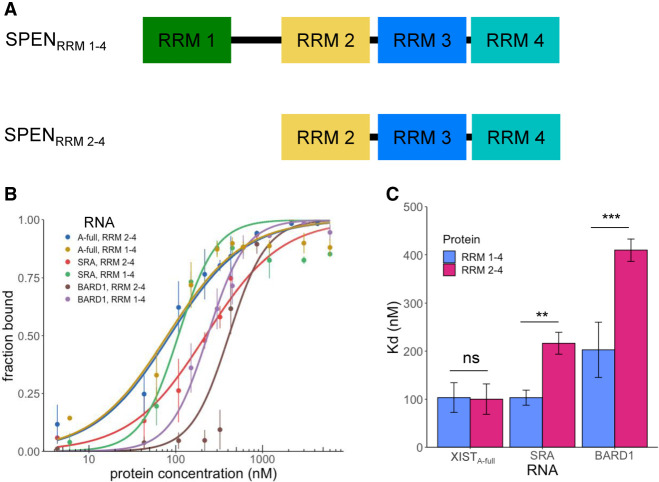
SPEN_RRM 1–4_ increases SRA RNA binding compared to SPEN_RRM 2–4_ construct. (*A*) Diagram of SPEN_RRM 1–4_ and SPEN_RRM 2–4_ protein constructs. (*B*) Quantification of the bound fraction of SPEN_RRM 1–4_ and SPEN_RRM 2–4_ with XIST_A-full_, SRA, or BARD1 RNA from EMSA experiments, *n* = 3–6. Data are the mean ± SEM. (*C*) Comparison of binding affinities for SPEN_RRM 1–4_ and SPEN_RRM 2–4_ with XIST_A-full_, SRA, or BARD1 RNA from replicate experiments, *n* = 3–6. (*) *P* < 0.05, (**) *P* < 0.01, (***) *P* < 0.001. Data are the mean ± SEM.

In previous work, the binding of a SPEN_RRM 2–4_ protein construct to SRA RNA was examined in detail in vitro (Arieti et al. 2014). We compared the contribution of RRM 1 and the linker sequence between RRM 1 and RRM 2 of SPEN protein to the binding affinity of SPEN_RRM 2–4_ for the XIST_A-full_ and SRA RNA transcripts. The SPEN RRM 1–RRM 2 linker consists of a low complexity, predicted intrinsically disordered protein region with multiple serine and arginine residues. Low-complexity protein domains have been proposed to confer nonspecific RNA binding (Basu and Bahadur 2016). SPEN_RRM 2–4_ is necessary for the initial recruitment of SPEN to the X chromosome while RRM 1 has been found to be dispensable for XCI (Dossin et al. 2020). Consistent with these results, we found that inclusion of RRM 1 in the SPEN_RRM 1–4_ construct had no effect on XIST_A-full_ binding. However, inclusion of this additional protein sequence significantly increased SPEN_RRM 1–4_ binding to both SRA RNA and the negative control BARD1 RNA, when compared to the previously studied SPEN_RRM 2–4_ construct containing only RRM 2, RRM 3, and RRM 4 (Fig. 2).

### SPEN RRM 3 is required for binding to SRA RNA but not to XIST_A-full_ RNA

**FIGURE 3.**
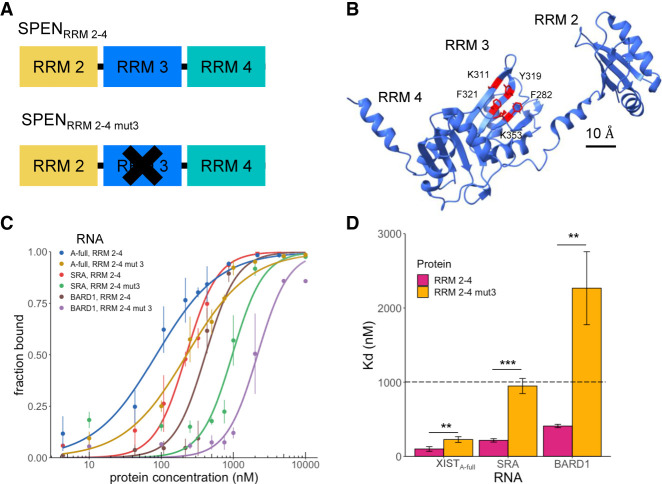
SPEN RRM 3 is required for binding to SRA RNA but not to XIST_A-full_ RNA. (*A*) Diagram of SPEN_RRM 2–4_ and the mutated RRM 3 protein construct SPEN_RRM 2–4 mut3_ used for RNA-binding studies. (*B*) Locations of mutations in the SPEN_RRM 2–4 mut3_ mutated RRM 3 construct which were mutated to alanine. (*C*) Quantification of the bound fraction of SPEN_RRM 2–4_ and SPEN_RRM 2–4 mut3_ with XIST_A-full_, SRA, or BARD1 RNA from EMSA experiments, *n* = 3. (SHARP_RRM 2–4_) or *n* = 4 (SHARP_RRM 2–4 mut3_). Data are the mean ± SEM. (*D*) Comparison of binding affinities for SPEN_RRM 2–4_ and SPEN_RRM 2–4 mut3_ for XIST_A-full_, SRA, or BARD1 RNA from replicate experiments, *n* = 3 (SPEN_RRM 2–4_) or *n* = 4 (SPEN_RRM 2–4 mut3_). (*) *P* < 0.05, (**) *P* < 0.01, (***) *P* < 0.001. Data are the mean ± SEM.

SRA binding to SPEN_RRM 2–4_ can be eliminated by mutation of several aromatic amino acid residues at the RNA-binding interface of RRM 3 within the SPEN_RRM 2–4_ protein construct (Arieti et al. 2014). We created the same RRM 3 mutant SPEN_RRM 2–4 mut3_ protein and tested whether the RRM 3 domain was similarly required for SPEN recognition of XIST RNA, or whether these two RNA targets can be recognized differently by the protein. The negative control for nonspecific RNA binding in these experiments was BARD1 mRNA, a size-matched fragment from an unrelated messenger RNA that would not be expected to bind to SPEN. Confirming the previous findings, SPEN_RRM 2–4 mut3_ binding to SRA was greatly reduced when RRM 3 residues were mutated, confirming that specific aromatic amino acid mutations (F282A, K311A, Y319A, F321A, and K353A) on the β-sheet face of RRM 3 disrupted SRA binding (Fig. 3). Mutation of aromatic residues in RRM 3 of SPEN_RRM 2­–4_ resulted in a loss of binding (defined as *K*_*d*_ > 1000 nM) for both SRA and the negative control, BARD1 RNA. Mutation of the aromatic residues of SPEN RRM 3 did not eliminate binding to XIST in the same manner as for SRA RNA and BARD1 RNA. Mutations in the construct SPEN_RRM 2–4 mut3_ decreased but did not completely abrogate binding to XIST_A-full_ A-repeat RNA, resulting in a twofold reduction of the *K*_*d*_ to 230 ± 40 nM (Table 2).

**TABLE 2.** *K*_*d*_ of XIST constructs and control RNA binding to SPEN_RRM 1–4_

RNA construct	*K*_*d*_ (nM)	Hill's coefficient
SRA	103 ± 16	2
XIST_A-full_	103 ± 31	1
XIST_A1–4_	71 ± 4	1
XIST_A4-6_	132 ± 10	2
XIST_A5-8_	127 ± 4	5
XIST_A6-9_	185 ± 9	2
XIST_A6-9+_	48 ± 5	1
XIST_A6-8_	288 ± 39	1
XIST_A7-8_	248 ± 68	1
BARD1 (negative control)	223 ± 54	2
tRNA_Cys_ (negative control)	245 ± 78	1

### SPEN RRM 4 is required for binding to any of the RNA transcripts examined

**FIGURE 4.**
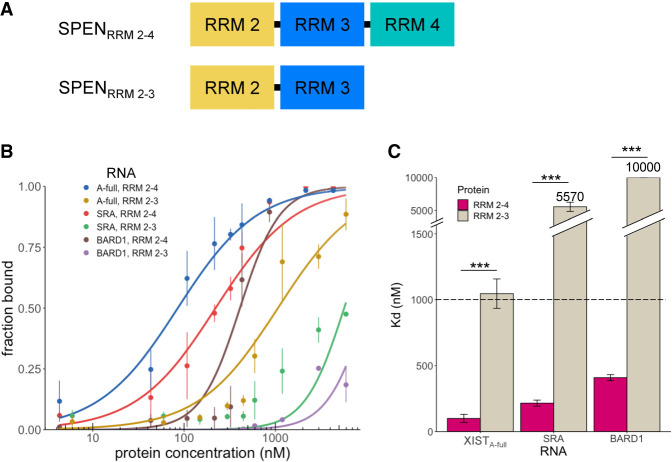
SPEN RRM 4 is required for binding to any of the RNA transcripts examined. (*A*) Diagram of SPEN_RRM 2–4_ and SPEN_RRM 2–3_ protein constructs. (*B*) Quantification of the bound fraction of SPEN_RRM 2–4_ and SPEN_RRM 2–3_ with XIST_A-full_, SRA, or BARD1 RNA from EMSA experiments, *n* = 3. Data are the mean ± SEM. (*C*) Comparison of binding affinities for SPEN_RRM 2–4_ and SPEN_RRM 2–3_ for experimental and control RNA targets from replicate experiments, *n* = 3. (*) *P* < 0.05, (**) *P* < 0.01, (***) *P* < 0.001. Data are the mean ± SEM.

Since mutations in RRM 3 did not eliminate SPEN binding to XIST, we next evaluated the contribution of RRM 4 to RNA-binding affinity and specificity. The minimal SRA RNA binding construct of SPEN is RRM 3–4 (Arieti et al. 2014), and our results showed that XIST and SRA bind mutually exclusively to SPEN_RRM 1–4_. Therefore, we hypothesized that RRM 4 would be required for both XIST binding and SRA binding. We found that RRM 4 was indeed required for XIST–SPEN or SRA–SPEN binding interactions (Fig. 4). The SPEN_RRM 2–3_ construct was unable to bind XIST or SRA with high affinity. Furthermore, elimination of SPEN RRM 4 to create the construct SPEN_RRM 2–3_ also resulted in a significant loss of binding to the negative control RNA transcript BARD1.

### Four XIST A-repeat units are sufficient for high-affinity SPEN_RRM 1–4_ binding

**FIGURE 5.**
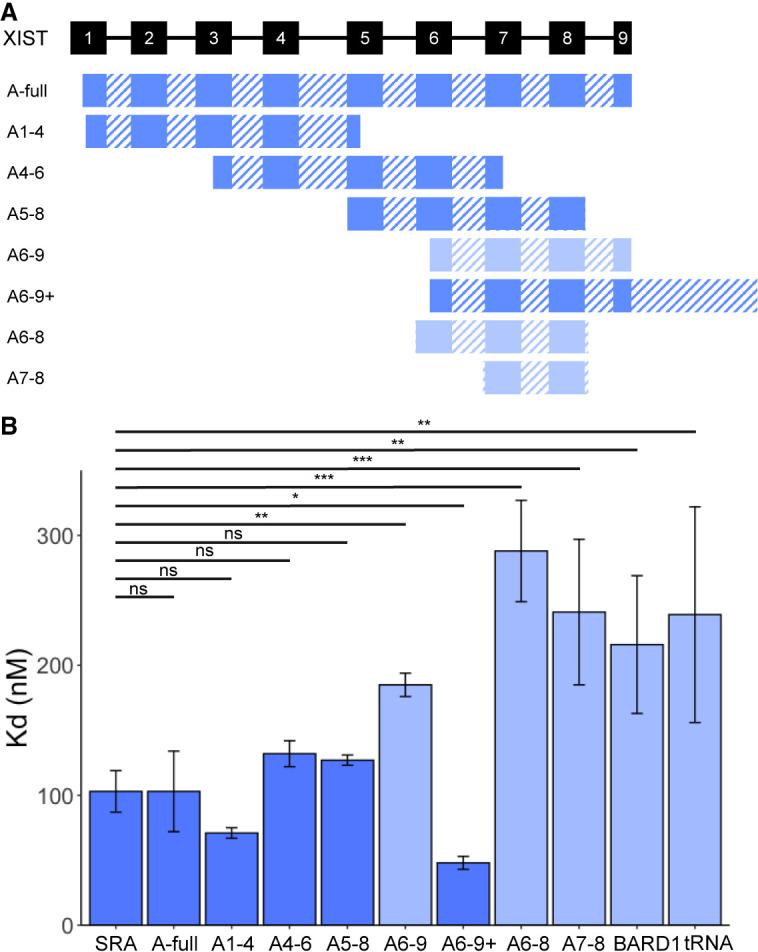
Four XIST A-repeat units are sufficient for high-affinity SPEN_RRM 1–4_ binding. (*A*) Diagram of XIST RNA constructs containing various A-repeat sequence units from repeats 1 through 9 (solid rectangles), and U-rich linker or additional sequences (hashed rectangles). (*B*) Comparison of binding affinities for SPEN_RRM 1–4_ to all XIST RNA constructs, SRA, BARD1, or tRNA_Cys_ negative controls. Dark blue color indicates high-affinity binding of the RNA construct to SPEN_RRM 1–4_. Light blue color indicates a significant decrease in SPEN–RNA binding compared to XIST_A-full_ transcript, *n* = 3–6. (*) *P* < 0.05, (**) *P* < 0.01, (***) *P* < 0.001. Data are the mean ± SEM.

To evaluate the minimum number of XIST A-repeat region RNA units required for binding to SPEN, we designed a series of RNA constructs with varying sequences of A-repeat units, linkers, and additional sequences in dimers, trimers, or multimers (Fig. 5A). Initial creation of RNA constructs was based on a structural model of the human XIST A-repeat region from previous work which combined enzymatic cleavage and chemical structure probing information (Maenner et al. 2010). The equilibrium dissociation constant of RNA binding to SPEN_RRM 1–4_ for each XIST A-repeat construct was measured and compared to XIST_A-full_ binding (Table 2). Negative controls for these in vitro experiments were the BARD1 RNA and a small, structured RNA transcript, tRNA_Cys_.

First, we determined whether dimer or trimer A-repeat sequences were sufficient for high-affinity SPEN_RRM 1–4_ binding. The trimer A-repeat RNA consisted of XIST A-repeat numbers 6–8 (XIST_A6–8_), while the dimer A-repeat RNA consisted of repeat numbers 7–8 (XIST_A7–8_). RNA transcripts containing two or three repeat units could not recapitulate the higher binding affinity of the XIST_A-full_ A-repeat region construct, and these RNA transcripts bound SPEN_RRM 1–4_ with similar affinity to the negative control transcripts (Fig. 5B; Table 2).

We next tested whether the identity of the specific XIST A-repeats included in the RNA transcript affected the RNA-binding affinity for SPEN_RRM 1–4_. The SPEN binding of three different XIST transcripts containing repeat unit numbers 1–4 (XIST_A1–4_), repeat numbers 4–6 (XIST_A4–6_), or repeat numbers 5–8 (XIST_A5–8_) were compared to the SPEN binding of the XIST_A-full_ RNA transcript containing all of the XIST repeats. We found that the exact identity of the repeat sequences included in each RNA construct did not significantly affect binding to SPEN_RRM 1–4_ (Fig. 5B). Since these three fragments were of similar length, but each contained portions of repeat number 5, we could not determine with certainty whether the presence of the sequence from repeat number 5 was enabling binding to SPEN_RRM 1–4_. Therefore, we cloned an RNA construct containing the A-repeat numbers 6–9, plus an additional region of the downstream XIST sequence (XIST_A6–9+_) to create an RNA of similar length to the previous three constructs. Surprisingly, the XIST_A6–9+_ RNA bound to SPEN_RRM 1–4_ with a significantly higher affinity than XIST_A-full_. Finally, a shorter RNA transcript, containing only the XIST A-repeat numbers 6–9, without the extended downstream sequence (XIST_A6–9_), showed much lower binding to SPEN_RRM 1–4_.

### SPEN binding changes XIST A-repeat accessibility to chemical probing

**FIGURE 6.**
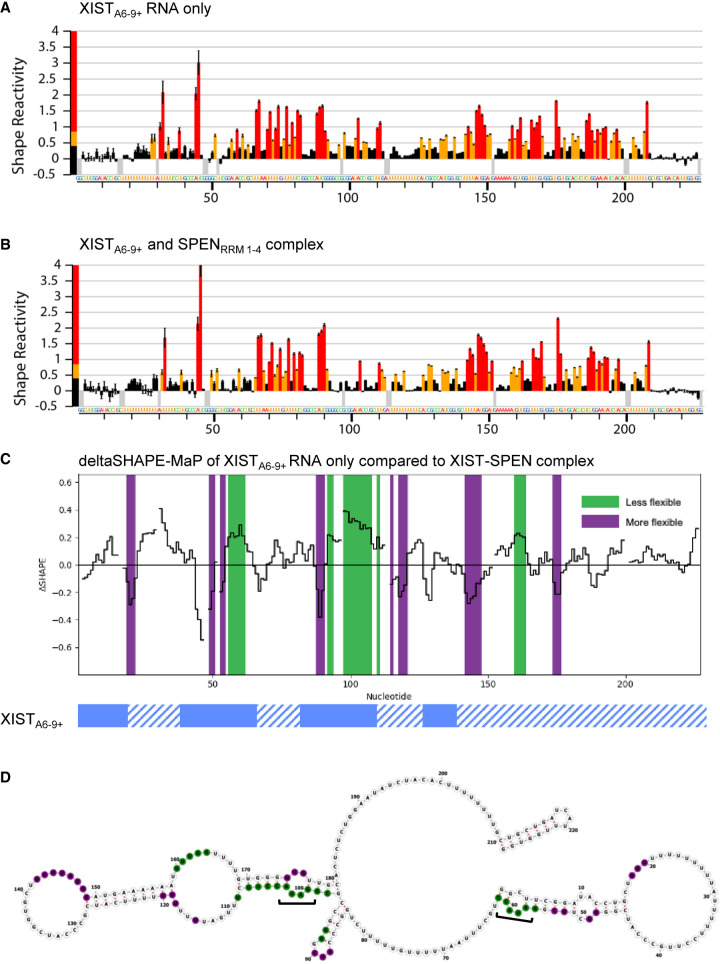
SPEN binding changes XIST A-repeat accessibility to chemical probing. SHAPE-MaP reactivity scores per nucleotide for (*A*) XIST_A6–9+_ RNA alone or (*B*) XIST_A6–9+_ RNA bound to SPEN_RRM 1–4_. (*C*) deltaSHAPE-MaP analysis of XIST RNA only compared to XIST–SPEN complex calculated from the two data sets above. Nucleotide positions colored in green showed decreased flexibility in the RNA–protein complex, while nucleotide positions colored in purple showed increased flexibility in the RNA–protein complex, compared to XIST_A6–9+_ RNA alone. (*D*) deltaSHAPE-MaP changes in nucleotide flexibility after SPEN binding were mapped onto the predicted secondary structure of XIST_A6–9+_ RNA alone. Nucleotide positions colored in green showed decreased flexibility in the RNA–protein complex, while nucleotide positions colored in purple showed increased flexibility in the RNA–protein complex, compared to XIST_A6–9+_ RNA alone. Unpaired adenosine sequences in the GAUAC sequence of the XIST A-repeat numbers 7 and 8 are indicated with brackets.

We next used chemical structure probing to examine changes in accessibility and reactivity of the XIST_A6–9+_ RNA transcript when bound or unbound to SPEN_RRM 1–4_. To this end, we performed SHAPE-MaP structural probing on the construct XIST_6–9+_ by treating the RNA with 1-methyl-7-nitrosatoic anhydride (1m7) either in the presence or absence of SPEN_RRM 1–4_. The RNA and protein concentrations for the SHAPE-MaP assay were selected to ensure complete binding of the RNA, based on the EMSA studies. Single nucleotide reactivity profiles were calculated for RNA only and RNA–protein complexes along the entire XIST_A6–9+_ transcript (Fig. 6A,B). Next, we calculated the deltaSHAPE-MaP for XIST RNA-only sample reactivity at each nucleotide position of the RNA, compared to the reactivity within the XIST–SPEN complex (Fig. 6C). The deltaSHAPE-MaP plot of flexible RNA bases revealed several changes in nucleotide accessibility upon protein binding. Notably, unpaired adenosines in a double-stranded RNA sequence were reduced in accessibility on both repeat 7 and repeat 8 of the XIST_A6–9+_ RNA transcript after SPEN_RRM 1–4_ binding (Fig. 6D). In both repeat 7 and repeat 8 structures, the unpaired nucleotides were the conserved adenosines in the GAUAC sequence in the 5′ region of the A-repeat unit. Repeat 6 also forms a similar structure with an unpaired adenosine but this sequence was included in the primer region, so we did not obtain nucleotide reactivity profiles in this area. Based on the secondary structure modeling combined with deltaSHAPE-MaP, inter-repeat interactions at the GAUAC sequence were most strongly altered upon protein binding.

### Presentation of an unpaired adenosine in a bulge or internal loop correlates with high-affinity SPEN binding

**FIGURE 7.**
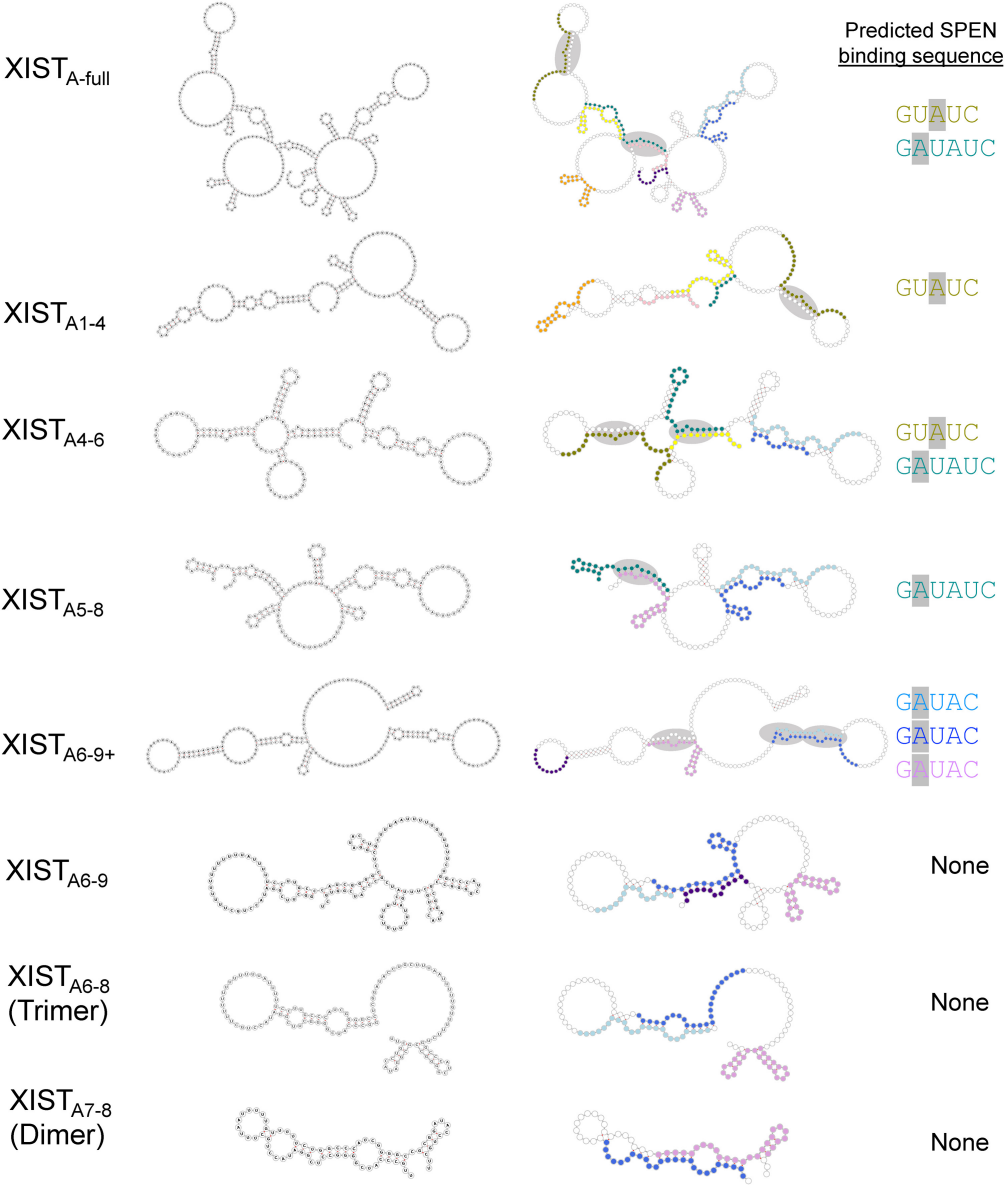
Presentation of an unpaired adenosine in a bulge or internal loop correlates with high-affinity SPEN binding. Minimum free energy secondary structure predictions for XIST A-repeat transcripts. RNA secondary structures are labeled with specific nucleotide sequences (*left* column) or colored according to the repeat number for XIST A-repeats 1–9 (*right* column). XIST A-repeat 1 is colored in pink, repeat 2 is orange, repeat 3 is yellow, repeat 4 is olive green, repeat 5 is dark green, repeat 6 is light blue, repeat 7 is dark blue, repeat 8 is violet, and repeat 9 is purple. Gray ovals indicate the location of unpaired adenosines in GUAUC, GAUAC, or GAUAUC sequences within a dsRNA context for each XIST A-repeat region transcript. The specific sequence (or sequences) containing an unpaired adenosine within an internal loop is color-coded by repeat number, and listed to the *right* of each secondary structure.

Secondary structure predictions of the minimum free energy conformations for each truncated A-repeat construct were analyzed for the presence of common RNA sequence and structure motifs to identify features correlated with high-affinity SPEN binding in vitro (Fig. 7). We particularly focused on the differences between XIST_A6–9_ and XIST_A6–9+_ since the latter RNA showed the highest binding affinity for SPEN_RRM 1–4_. An unpaired adenosine in the GAUAC sequence of the A-repeat, presented in a bulge or small internal loop within the context of a double-stranded RNA helix, was identified in each repeat in the computationally predicted secondary structure of the extended XIST_A6–9+_ transcript when compared to XIST_A6–9_. Similarly, the other tested XIST RNA transcripts containing an unpaired adenosine in one or more G**A**UAC, GU**A**UC, or G**A**UAUC sequence, when presented within a bulge or small internal loop (XIST_A-full_, XIST_A1–4_, XIST_A4–6_, and XIST_A5–8_) were able to bind SPEN_RRM 1–4_ with similar high affinity in the in vitro binding assays (Fig. 7, gray ovals). A statistically significant decrease in the in vitro binding affinity for SPEN_RRM 1–4_ was observed for XIST A-repeat transcripts XIST_A6–9_, XIST_A6–8_ (trimer), and XIST_A7–8_ (dimer) which did not present this specific combination of sequence and structural motifs.

## DISCUSSION

In this study, we dissected the regions required for RNA interaction and protein binding in the XIST–SPEN complex through in vitro binding experiments and RNA structure probing. This RNA–protein interaction is necessary for the initiation of X chromosome silencing in humans. We constructed a series of mutants of human SPEN including RRM 1–RRM 4 and quantified the affinity of these proteins for binding to SRA, XIST, or negative control RNA transcripts (Table 1). SPEN_RRM 1–4_ bound strongly to either SRA or the XIST A-repeat RNA region (XIST_A-full_) with a similar *K*_*d*_ of 100 nM for each RNA transcript. Binding of SPEN_RRM 1–4_ to a size-matched negative control transcript from an unrelated messenger RNA (BARD1) or tRNA_Cys_ showed a significantly higher *K*_*d*_ around 250–300 nM. A competitive EMSA of SPEN_RRM 1–4_ binding to SRA and XIST showed that SRA was able to outcompete XIST_A-full_ binding to SPEN. The negative control BARD1 RNA could not compete with XIST_A-full_ for SPEN binding.

Since SRA and XIST RNA are both functional binding partners of SPEN in human cells, we investigated the determinants of these RNA–protein interactions by mutation or deletion of SPEN RRMs predicted to be required for RNA binding. SPEN contains four RRM domains. Previous work focused on the functions of SPEN RRM 2, RRM 3, and RRM 4 in SRA binding. We found that inclusion of RRM 1 increased the binding affinity of SPEN for both SRA and the negative control RNA BARD1. However, inclusion of the SPEN RRM 1 domain did not increase the binding affinity of the protein for the XIST A-repeat region RNA. We concluded that the RRM 1 domain is not required for high-affinity XIST A-repeat region binding in vitro but increases SPEN binding to the other RNA transcripts tested. Although XIST and SRA bind SPEN_RRM 1–4_ in a mutually exclusive manner, the molecular details of the interactions between SPEN and these two RNA transcripts are not identical.

Next, we investigated whether the same RRM domains of SPEN were required for both SRA and XIST binding. Previously, SPEN RRM 3 and RRM 4 were both shown to be required for specific binding to human SRA RNA. Binding depends on both single and double-stranded regions of SRA (Hatchell et al. 2006; Arieti et al. 2014). Mutation of SPEN RRM 3 or RRM 4 domains had different effects on binding to the XIST or SRA RNA transcripts. In contrast to SRA binding, we observed that the canonical SPEN RRM 3 RNA-binding residues were not required to maintain high-affinity interactions with the XIST A-repeat region RNA in vitro. These results were consistent with the previous finding that RRM 3 mutation results in an approximately twofold decrease in binding to XIST A-repeat by fluorescence anisotropy (Carter et al. 2020). The SPEN_RRM 2–3_ construct was unable to bind XIST or SRA with high affinity, so we concluded that a functional RRM 4 was required for binding to XIST A-repeat region RNA. The combination of RRM 2, mutated RRM 3, and RRM 4 did retain some additional XIST binding compared to the SPEN_RRM 2–4_ construct, so it is possible that the combination of RRM 2 and RRM 4 or the linkers between these regions may play a role in binding XIST. Additional experiments with specific mutation or deletion of residues in RRM 2 would be required to assess the contribution of RRM 2 to SPEN–XIST binding. Loss of RRM 4 also destroyed binding to all other RNA transcripts tested. In the SPEN_RRM 2–4_ crystal structure, a C-terminal helix occludes the β-sheet face of RRM 4 (Arieti et al. 2014). Atypical RRM domains with structures similar to SPEN RRM 4 have also been identified in other RBPs including Prp24 (Montemayor et al. 2014) and U1A (de Vries et al. 2022).

We also investigated the minimal number of XIST A-repeats required for high-affinity binding between SPEN and XIST in vitro. We found that at least four full or partial XIST RNA A-repeat sequences were required for high-affinity RNA–protein interactions with SPEN_RRM 1–4_. Human XIST contains eight and a half A-repeat sequences, numbered here as repeats 1–9 for simplicity, beginning from the 5′ end of the RNA. In human XIST, nucleotide sequence variation occurs across individual repeats to give each repeat a distinct sequence identity (Supplemental Fig. S1). Four different XIST A-repeat constructs of similar length were created that each contained a set of four or more repeats: XIST_A1–4_, XIST_A4–6_, XIST_A5–8_, and XIST_A6–9+_. The linear sequence length of the fragment containing XIST A-repeat transcripts did not directly correlate with the in vitro binding affinity for SPEN. Each of these transcripts bound SPEN_RRM 1–4_ with similar or higher affinity than the XIST_A-full_ RNA containing the full complement of human A-repeats.

Surprisingly, we found that the RNA transcript with the highest binding affinity for SPEN_RRM 1–4_ was the XIST_A6–9+_ construct comprised of a partial repeat 6, full repeats 7 and 8, and the partial repeat 9 plus additional RNA sequence downstream from the A-repeat region. Truncation of the additional downstream sequence of XIST_A6–9+_ to create a transcript consisting of only the terminal repeats (XIST_A6–9_) resulted in loss of binding to SPEN_RRM 1–4._ This transcript had a binding affinity similar to the nonspecific negative control RNA transcripts BARD1 and tRNA_Cys_. However, this specific downstream sequence does not appear to be required for high-affinity binding. The XIST_A5–8_ construct containing four complete A-repeats was also able to recover high-affinity binding to SPEN. Four-repeat structures of XIST RNA have been predicted to have low free energies and can contain functional inter-repeat interactions, based on previous work (Maenner et al. 2010).

To identify potential protein interaction regions, we created a secondary structure model of XIST_A6–9+_ based on SHAPE-MaP structure probing in the presence or absence of SPEN_RRM 1–4_. The per-nucleotide reactivity and flexibility of XIST_A6–9+_ RNA were calculated both with and without binding to the SPEN_RRM 1–4_ protein. Notably, unpaired adenosines in a double-stranded helical context in the GAUAC sequences on both repeat 7 and repeat 8 in our XIST_A6–9+_ model had reduced flexibility upon SPEN binding. We compared the sequence and structural features of the computationally predicted minimum free energy RNA secondary structures of each A-repeat construct. We particularly focused on differences between the XIST_A6–9_ and XIST_A6–9+_ RNA transcripts, since these two constructs contained similar sequence information but showed highly divergent in vitro SPEN binding affinities. The comparative analysis of computationally predicted RNA secondary structures based on sequence information revealed that the presence of a single adenosine in an unpaired bulge or small internal loop in a G**A**UAC, GU**A**UC, or G**A**UAUC sequence was correlated with high SPEN binding affinity of the RNA transcript in vitro (Fig. 7). A major difference between XIST_A6–9+_ and XIST_A6–9_ is the ability to present this specific combination of sequence and structural motifs. The additional downstream nonrepeat sequences included in the strongly binding XIST_A6–9+_ RNA can interact through G-U wobble pairs with the 5′ portion of repeat 8. These interactions create a “pseudo inter-repeat” structure to enable the presentation of an unpaired adenosine in the GAUAC sequence. In our SHAPE studies of XIST_A6–9+_, the GAUAC sequences in both repeat 7 and repeat 8 were significantly reduced in flexibility when bound by SPEN_RRM 1–4_. A significantly lower SPEN binding affinity was observed for XIST_A6–9_ RNA, where this combination of sequence and structural motifs is not present.

The binding data combined with the computational secondary structure predictions suggest that high-affinity XIST–SPEN interactions may be facilitated by the presentation of an unpaired adenosine in a GAUAC or similar sequence, within a bulge or small internal loop in a double-stranded RNA context. A minimum of four full or partial A-repeat sequences were required for high-affinity SPEN binding in vitro, and a significant loss in SPEN binding for XIST RNA was observed in transcripts which did not have this combination of sequence and structural motifs in the computationally predicted secondary structures based on sequence information. A dimer of A-repeats (XIST_A7–8_) or trimer of A-repeats (XIST_A6–8_) showed significantly lower binding affinity for SPEN_RRM 1–4_ (*K*_*d*_ ∼300 nM), similar to the negative controls. RNA transcripts with higher numbers of A-repeats may be able to access multiple inter-repeat conformations that would not be possible to create with a dimer repeat construct, trimer repeat construct, or the shorter XIST_A6–9_ construct. In several constructs, repeat 4 was predicted to form noncanonical interactions with portions of the adjacent U-rich linkers. Although the U-rich linkers are overall less highly conserved than the repeat regions, they may still contribute to the XIST A-repeat region function (Liu et al. 2017). The negative control RNA transcripts tRNA_Cys_ and BARD1 do not contain unpaired adenosines in a GAUAC or similar sequence in an extended stem–loop structure in the computationally predicted secondary structures. This pattern along with our SHAPE data suggests that SPEN binding to XIST A-repeat RNA could be facilitated by a combination of minimal sequence and structural components that can be formed by the conserved A-repeat units as well as regions of the linkers and downstream sequences.

We compared our results with other models of XIST A-repeat RNA structure and SPEN binding and observed several similarities with the previous results. Previous structural studies indicated that the conserved D1 domain of XIST RepA, which contains the A-repeat region, folds as an independent unit (Liu et al. 2017). A pairwise alignment of RNA secondary structure with BEAGLE (Mattei et al. 2015) revealed a significant correlation between the secondary structure of our XIST_A6–9+_ model and the same RNA region from a model of the entire XIST RNA based on a prior in-cell SHAPE-seq data set (Sun et al. 2019; Carter et al. 2020). The RNA models showed around 30% structural similarity, with an alignment *P-*value of 0.003, indicating that secondary structures formed by XIST_A6–9+_ in vitro are similar to the structures formed by the same region in the full-length XIST RNA in vivo.

Each XIST A-repeat contains two regions of high sequence conservation: a 5′ region with a CAUCG sequence and a 3′ region with a GAUAC sequence (Supplemental Fig. S1). Each repeat 1–9 also has an individual sequence identity based on nucleotide variation outside of these two conserved regions (Supplemental Fig. S1). The ability of A-repeat sequences to form thermodynamically stable AUCG tetraloops in the 5′ region and a propensity for the 3′ region containing the GAUAC sequence to create inter-repeat interactions was previously characterized by NMR (Duszczyk et al. 2011). Focusing on SPEN binding, only a fraction of XIST transcripts in the cell may be bound during in vivo structure probing experiments and a large number of A-repeat secondary structure conformations and combinations may exist simultaneously in cells. Our data on changes in the in vitro structure of the XIST RNA upon SPEN binding, and direct comparison of changes among specific mutants of XIST A-repeat RNA, have therefore enabled additional insights into the sequences and structures required for this specific RNA–protein binding interaction that are complementary to the observations obtained from in-cell experiments. The deltaSHAPE-MaP data indicate that the largest changes in nucleotide accessibility upon SPEN binding to XIST occurred in regions with a bulged adenosine in the conserved GAUAC sequence, when this sequence was presented within a double-stranded context in an inter-repeat interaction. In some models of XIST A-repeat structure, GAUAC sequences are unpaired and form part of a large loop along with an adjacent U-rich linker sequence, or alternately, form a short stem–loop (Maenner et al. 2010; Fang et al. 2015; Liu et al. 2017; Carter et al. 2020). In CLIP experiments, the GAUAC sequence in the XIST A-repeat also showed the highest crosslinking signal after capture of the endogenous SPEN protein, indicating that this RNA region is likely to be important for SPEN binding in cells (Carter et al. 2020). Our results are consistent with the hypothesis that a specific type of inter-repeat interaction by XIST repeats is required to occur in order to present the appropriate combination of sequence and structural information for SPEN binding. NMR studies of XIST A-repeat dimers showed a potential for inter-repeat interactions to create an unpaired adenosine structure in the GAUAC sequence (Duszczyk et al. 2011). In our in vitro studies, XIST A-repeat structures predicted by minimum free energy computational secondary structure modeling to contain this specific combination of structure and sequence motifs also showed high-affinity SPEN binding. Inter-repeat interactions have been found in living cells, and complex inter-repeat interactions in the XIST RNA structure may create multiple simultaneously coexisting RNA structures in vivo (Lu et al. 2016). In addition, multiple tertiary structures have been described for the RepA transcript which contains the A-repeat region (Aguilar et al. 2022). A novel insight from our in vitro studies was that the identity of the XIST repeats included in each construct did not significantly affect SPEN binding interactions in vitro. Given that the specific identity of repeats included in each XIST construct did not impact the in vitro RNA-binding affinity for SPEN_RRM 1–4_, we hypothesized that multiple combinations of inter-repeat interactions may be able to present an unpaired adenosine within an internal loop of the GAUAC or similar sequence to facilitate high-affinity SPEN binding.

These findings are consistent with previous studies of the in vivo functions of truncated or synthetic XIST A-repeat constructs in mouse and human cells, while providing novel insight on the specific sequence and structural information that may facilitate high-affinity SPEN binding. Constructs containing fewer than eight and a half A-repeats can still achieve silencing of a nearby transgene by XIST in human cells, though with much lower efficacy as repeat numbers decrease (Minks et al. 2013). Similarly, four synthetic consensus “XCR” repeats could still accomplish a low level of silencing on the X chromosome (Wutz et al. 2002). XIST may have co-opted the existing nucleic acid binding ability of SPEN to achieve developmentally regulated gene silencing (Elisaphenko et al. 2008; Carter et al. 2020), since SPEN proteins can interact with multiple functional RNA transcripts. These proteins can have multiple binding sites on autosomes in addition to interaction sites across the X chromosome (Dossin et al. 2020). Since XIST can effectively compete with SRA and other RNA transcripts for SPEN binding, unique binding modes for each RNA may enable precisely timed XIST RNA expression in development to enable recruitment of SPEN to the X chromosome during silencing initiation. Since our binding experiments were performed in vitro with unmodified RNA transcripts, it is not possible to evaluate the contribution of RNA modifications on XIST to SPEN binding. Further research will be needed to validate the physiological relevance of these findings and verify whether the presentation of an unpaired adenosine in the context of a double-stranded inter-repeat interaction can contribute to the binding of XIST and SPEN during XCI in human cells.

Evolutionary modulation of RNA and RBP interactions can occur through the incorporation of multiple protein domains (Lunde et al. 2007). In the case of SPEN, the presence of multiple RRM domains with different nucleotide binding specificities may allow the protein to achieve transient binding in the nuclear environment, while still preserving the ability to interact with specific RNA transcripts including SRA and XIST during development. Similarly, the inclusion of multiple XIST A-repeat sequences may enable flexibility and protection against insertions, deletions, or mutations, since only four repeats are required to form the correct presentation of sequence and structural motifs to facilitate high-affinity SPEN binding. In conclusion, this work provides insight into the complex interactions between XIST and SPEN, highlighting the contributions of different regions of the protein and RNA to forming a biologically critical interaction driving gene silencing in early female development.

## MATERIALS AND METHODS

### Cloning and purification of SPEN_RRM 1–4_ and mutant proteins

Nucleotide sequence encoding human SPEN_RRM 1–4_ was cloned from HEK293 complementary DNA (cDNA) prepared from purified total RNA by reverse transcription. A 6XHistidine tag, Maltose binding protein, and a TEV cleavage site were added to the N-terminus of the protein. Point mutations were created using gene synthesis. The SPEN RRM 3 mutant constructs contained the following mutations: F282A, K311A, Y319A, F321A, and K353A. SPEN protein purifications were performed essentially following the method of Arieti et al. (2014), with some modifications as listed below. All proteins were produced with the same method. First, *Escherichia coli* Rosetta 2 cells transformed with the construct of interest were grown in Terrific Broth medium at 37°C until ∼0.4 OD_600_, followed by overnight induction at 16°C with 1 mM IPTG and 0.1% arabinose. *E. coli* were harvested by centrifugation at 1000*g* for 60 min, washed with 1× PBS, and then resuspended in 50 mM HEPES buffer, pH 7.5 containing 300 mM NaCl, 20 mM imidazole, 0.1% Triton-X100, 1 μg/mL DNase I, 1 μg/mL lysozyme, 5 mM β-mercaptoethanol, and 1× Roche cOmplete protease inhibitor (EDTA-free). After a 30-min incubation on ice, cells were sonicated using a Branson microtip, and the lysate was cleared by centrifugation at 10,000*g* for 20 min. The clarified lysate was then applied to a 10 mL Ni Sepharose HP column (17-5268-02 GE). The protein was eluted with 250 mM imidazole and dialyzed against 50 mM HEPES (pH 7.5), 300 mM NaCl, 20 mM imidazole, and 5 mM β-mercaptoethanol overnight. TEV protease was added, and the sample was incubated at 30°C for 2 h. This sample was then cleared with centrifugation at 10,000*g* for 20 min and the supernatant was applied to charged Ni-NTA resin again. The unbound material from this column was collected, diluted twofold with heparin binding buffer (50 mM HEPES [pH 7], 0.1% Triton-X, 5 mM β-mercaptoethanol) and then applied to a 2 mL heparin column (Hi-Trap Heparin HP GE). Elution was performed with a linear salt gradient between 0.05 and 2 M of NaCl. The protein was further purified by gel filtration chromatography with a HiLoad 16/600 Superdex 200 pg (28-9893-35, GE Healthcare) column in 50 mM HEPES (pH 7.5) containing 300 mM NaCl, 5 mM β-mercaptoethanol, 0.1% Triton-X. The protein eluted from the gel filtration column as monomers and was concentrated with a 10 kDa MWCO Amicon spin filter to ∼10 mg/mL. All protein samples were more than 95% pure as judged by Coomassie-stained SDS–PAGE. Sequences of the protein constructs used in this study are provided in Table 3. Purified bovine serum albumin (BSA) protein negative control was purchased from Sigma-Aldrich.

**TABLE 3.** Sequences of protein and RNA constructs used in this study.

SPEN_RRM 1–4_	MVRETRHLWVGNLPENVREEKIIEHFKRYGRVESVKILPKRGSEGGVAAFVDFVDIKSAQKAHNSVNKMGDRDLRTDYNEPGTIPSAARGLDDTVSIASRSREVSGFRGGGGGPAYGPPPSLHAREGRYERRLDGSDSSSSSSDDSPARSVQSAAVPAPTSQLLSSLEKDEPRKSFGIKVQNLPVRSTDTSLKDGLFHEFKKFGKVTSVQIHGTSEERYGLVFFRQQEDQEKALTASKGKLFFGMQIEVTAWIGPETESENEFRPLDERIDEFHPKATRTLFIGNLEKTTTYHDLRNIFQRFGEIVDIDIKKVNGVPQYAFLQYCDIASVCKAIKKMDGEYLGNNRLKLGFGKSMPTNCVWLDGLSSNVSDQYLTRHFCRYGPVVKVVFDRLKGMALVLYNEIEYAQAAVKETKGRKIGGNKIKVDFANRESQLAFYHCMEKSGQDIRDFYEMLAERREER
SPEN_RRM 2–4_	FGIKVQNLPVRSTDTSLKDGLFHEFKKFGKVTSVQIHGTSEERYGLVFFRQQEDQEKALTASKGKLFFGMQIEVTAWIGPETESENEFRPLDERIDEFHPKATRTLFIGNLEKTTTYHDLRNIFQRFGEIVDIDIKKVNGVPQYAFLQYCDIASVCKAIKKMDGEYLGNNRLKLGFGKSMPTNCVWLDGLSSNVSDQYLTRHFCRYGPVVKVVFDRLKGMALVLYNEIEYAQAAVKETKGRKIGGNKIKVDFANRESQLAFYHCMEKSGQDIRDFYEMLAERREER
SPEN_RRM 3–4_	RTLFIGNLEKTTTYHDLRNIFQRFGEIVDIDIKKVNGVPQYAFLQYCDIASVCKAIKKMDGEYLGNNRLKLGFGKSMPTNCVWLDGLSSNVSDQYLTRHFCRYGPVVKVVFDRLKGMALVLYNEIEYAQAAVKETKGRKIGGNKIKVDFANRESQLAFYHCMEKSGQDIRDFYEMLAERREER
SPEN_RRM 2–4 mut3_	FGIKVQNLPVRSTDTSLKDGLFHEFKKFGKVTSVQIHGTSEERYGLVFFRQQEDQEKALTASKGKLFFGMQIEVTAWIGPETESENEFRPLDERIDEFHPKATRTL**A**IGNLEKTTTYHDLRNIFQRFGEIVDIDI**A**KVNGVPQ**A**A**A**LQYCDIASVCKAIKKMDGEYLGNNRLKLGFG**A**SMPTNCVWLDGLSSNVSDQYLTRHFCRYGPVVKVVFDRLKGMALVLYNEIEYAQAAVKETKGRKIGGNKIKVDFANRESQLAFYHCMEKSGQDIRDFYEMLAERREER
XIST_A-full_	GGGCUGCGGAUACCUGGUUUUAUUAUUUUUUCUUUGCCCAACGGGGCCGUGGAUACCUGCCUUUUAAUUCUUUUUUAUUCGCCCAUCGGGGCCGCGGAUACCUGCUUUUUAUUUUUUUUUCCUUAGCCCAUCGGGGUAUCGGAUACCUGCUGAUUCCCUUCCCCUCUGAACCCCCAACACUCUGGCCCAUCGGGGUGACGGAUAUCUGCUUUUUAAAAAUUUUCUUUUUUUGGCCCAUCGGGGCUUCGGAUACCUGCUUUUUUUUUUUUUAUUUUUCCUUGCCCAUCGGGGCCUCGGAUACCUGCUUUAAUUUUUGUUUUUCUGGCCCAUCGGGGCCGCGGAUACCUGCUUUGAUUUUUUUUUUUCAUCGCCCAUCGGUGCU
XIST_A1–4_	GCUGCGGAUACCUGGUUUUAUUAUUUUUUCUUUGCCCAACGGGGCCGUGGAUACCUGCCUUUUAAUUCUUUUUUAUUCGCCCAUCGGGGCCGCGGAUACCUGCUUUUUAUUUUUUUUUCCUUAGCCCAUCGGGGUAUCGGAUACCUGCUGAUUCCCUUCCCCUCUGAACCCCCAACACUCUGGCCCAUCG
XIST_A4–6_	GCCGCGGAUACCUGCUUUUUAUUUUUUUUUCCUUAGCCCAUCGGGGUAUCGGAUACCUGCUGAUUCCCUUCCCCUCUGAACCCCCAACACUCUGGCCCAUCGGGGUGACGGAUAUCUGCUUUUUAAAAAUUUUCUUUUUUUGGCCCAUCGGGGCUUCGGAUACCUGCUUUUUUUUUUUUUAUUUUUCCUUGCCCAUCGGGG
XIST_A5–8_	GCCCAUCGGGGUGACGGAUAUCUGCUUUUUAAAAAUUUUCUUUUUUUGGCCCAUCGGGGCUUCGGAUACCUGCUUUUUUUUUUUUUAUUUUUCCUUGCCCAUCGGGGCCUCGGAUACCUGCUUUAAUUUUUGUUUUUCUGGCCCAUCGGGGCCGCGGAUACCUGCUU
XIST_A6–8_	GGCCCAUCGGGGCUUCGGAUACCUGCUUUUUUUUUUUUUAUUUUUCCUUGCCCAUCGGGGCCUCGGAUACCUGCUUUAAUUUUUGUUUUUCUGGCCCAUCGGGGCCGCGGAUACCUGCUU
XIST_A7–8_	GUUGCCCAUCGGGGCCUCGGAUACCUGCUUUAAUUUUUGUUUUUCUGGCCCAUCGGGGCCGCGGAUACCUGCUU
XIST_A6–9_	GGCUUCGGAUACCUGCUUUUUUUUUUUUUAUUUUUCCUUGCCCAUCGGGGCCUCGGAUACCUGCUUUAAUUUUUGUUUUUCUGGCCCAUCGGGGCCGCGGAUACCUGCUUUGAUUUUUUUUUUUCAUCGCCCAUCGGUGCU
XIST_A6–9+_	GGCUUCGGAUACCUGCUUUUUUUUUUUUUAUUUUUCCUUGCCCAUCGGGGCCUCGGAUACCUGCUUUAAUUUUUGUUUUUCUGGCCCAUCGGGGCCGCGGAUACCUGCUUUGAUUUUUUUUUUUCAUCGCCCAUCGGUGCUUUUUAUGGAUGAAAAAAUGUUGGUUUUGUGGGUUGUUGCACUCUCUGGAAUAUCUACACUUUUUUUUGCUGCUGAUCAUUUGGUGG
SRA	GUACCAGGUAUGUGAUGACAUCAGCCGACGCCUGGCACUGCUGCAGGAACAGUGGGCUGGAGGAAAGUUGUCAAUACCUGUAAAGAAGAGAAUGGCUCUACUGGUGCAAGAGCUUUCAAGCCACCGGUGGGACGCAGCAGAUGACAUCCACCGCUCCCUCAUGGUUGACCAUGUGACUGAGGUCAGUCAGUGGAUGGUAGGAGUUAAAAGAUUAAUUGCAGAAAAGAGGAGUCUGUUUUCAGAGGAGGCAGCCAAUGAAGAGAAAUCUGCAGCCACAGCUGAGAAGAACCAUACCAUACCAGGCUUCCAGCAGGCUUCAUAAUCCUCGGUUCCCCAGACUCACCGGACACCAUCUCCU
BARD1	GCGAGGAGCCUUUCAUCCGAAGGCGGGACGAUGCCGGAUAAUCGGCAGCCGAGGAACCGGCAGCCGAGGAUCCGCUCCGGGAACGAGCCUCGUUCCGCGCCCGCCAUGGAACCGGAUGGUCGCGGUGCCUGGGCCCACAGUCGCGCCGCGCUCGACCGCCUGGAGAAGCUGCUGCGCUGCUCGCGUUGUACUAACAUUCUGAGAGAGCCUGUGUGUUUAGGAGGAA
tRNA_Cys_	GGGGGUAUAGCUCAGGGGUAGAGCAUUUGACUGCAAAUCAAGAGGUCCCUGAUUCAAAUCCAGGUGCCCCCUCCAAGCUUG

### Cloning and production of XIST A-repeat RNA transcripts

XIST A-repeat RNA was cloned from human cDNA prepared from purified total RNA by reverse transcription. Primers were designed to target specific repeats or partial repeats and provide amplification specificity in the repeat region. All clones were validated by Sanger Sequencing through Azenta. Plasmid templates were linearized by restriction digestion, and RNA transcripts of interest were generated by in vitro runoff transcription using an NEB HiScribe T7 High Yield RNA synthesis kit (E2040S), with the addition of fluorophore-labeled UTPs: Cyanine 3-uridine-5′-triphosphate (enhanced) from Enzo Life Sciences, Inc. (catalog #ENZ-42505), fluorescein-12-uridine-5′-triphosphate from Enzo Life Sciences, Inc. (catalog #ENZ-42834), or cyanine 5-UTP from Apexbio Technology LLC (purchased through Fisher Scientific catalog #50-199-8343). In vitro transcribed RNA was purified with Zymo Clean & Concentrator-5 Kit. RNA transcript integrity and complexity were evaluated by gel electrophoresis or Agilent TapeStation analysis. Sequences of RNA constructs used in this study are provided in Table 3.

### Electrophoretic mobility shift assays

RNA transcripts with unique fluorophores were diluted to 2 nM each in UltraPure water and were denatured at 85°C for 5 min followed by rapid cooling on ice. Purified SPEN proteins were incubated with labeled RNA for 30 min at room temperature in buffer conditions containing 20 mM Tris-HCl (pH 7.5), 150 mM KCl, 5 mM MgCl_2_, and 10% glycerol and then loaded onto a 4.5% TBE polyacrylamide gel for separation of monomers and complexes. Multiplexed EMSA gels were imaged on a Typhoon FLA 9500 with a pixel size of 100 µm for each fluorophore. The laser/filter combinations were 473 nm/BPB1 for fluorescein, 532 nm/BPG1 for Cy3, and 635 nm/LPR for Cy5. Between three and six replicate experiments were performed for each RNA–protein combination, and the intensity of bands on each gel image was quantified using Image Studio Lite with background correction. The equilibrium dissociation constant (*K*_*d*_) for each reaction was calculated by fitting to the curve using the equation:

$$\mathrm{Fraction}\;\mathrm{bound}\;=\;\frac{{\left[P\right]}^{n}}{{\left[P\right]}^{n}+K_{d}}.$$

The custom R code used for *K*_*d*_ calculation from EMSA results was:

1. #DATA FROM EMSA
2. emsa.data <- data.frame(nM_protein_concentration = c (1, 10, 100, 250, 500, 10^3, 10^4),
3. fraction_bound = c(0,0.02,0.10,0.25,0.65,0.98, 1), stringsAsFactors = FALSE)
4. #LOGISTIC CURVE FIT
5. logistic_fit <- nls(data = emsa.data,
6. fraction_bound ∼ 1/(1 + (K_d/nM_protein_concentration)^n),
7. start = c(K_d = 10,n = 1), algorithm = “port”, lower = 0.001, upper =10000)
8. summary(logistic_fit)

Competitive EMSAs were performed as described in Carter et al. (2020). Briefly, Cy3 labeled XIST_A-full_ and SPEN_RRM 1–4_ concentrations were kept at 1 µM and 10 µM, respectively. Increasing concentrations of unlabeled competitor RNA were added. Competition reactions were done in buffer conditions containing 20 mM Tris-HCl (pH 7.5), 150 mM KCl, 5 mM MgCl_2_, and 10% glycerol, incubated at room temperature for 30 min and run on a 4.5% TBE polyacrylamide gel. The gels were imaged on a Typhoon FLA 9500 at 100 µM resolution using the laser/filter combination of 532 nm/BPG1 for Cy3.

### SHAPE-MaP reactions, high-throughput sequencing, and data analysis

The SHAPE-MaP protocol was adapted from previously published protocols (Smola and Weeks 2018). We followed the small RNA workflow with the addition of NEBNext mRNA second strand synthesis following cDNA synthesis. SHAPE-MaP of XIST_A6–9+_ RNA was performed either in the presence or absence of 1.7 µM SPEN_RRM 1–4_. After sequencing with MiSeq Micro at the UCSD Institute for Genomic Medicine, the SHAPE data were analyzed with ShapeMapper 2 (Busan and Weeks 2018). Changes in the nucleotide reactivity of XIST_A6–9+_ upon the binding of SPEN_RRM 1–4_ were calculated with deltaSHAPE_v1.0 (Smola and Weeks 2018).

### Sequence alignments and prediction of RNA secondary structures

Minimum free energy predictions of RNA secondary structures were calculated using the Vienna RNA Package programs RNAfold and visualized with forna (Gruber et al. 2008) or VARNA (Darty et al. 2009), using parameters as previously described (Mathews et al. 2004). For XIST_A6–9+_, the per-nucleotide reactivity data from SHAPE data was used in addition to RNA primary sequence; for all other constructs the primary RNA sequence was used to predict the secondary structure. Multiple sequence alignments were performed with MAFFT (Katoh et al. 2002) and visualized with MView (Madeira et al. 2022).   

## SUPPLEMENTAL MATERIAL

Supplemental material is available for this article.

## Figures and Tables

**Aileen C. Button:**
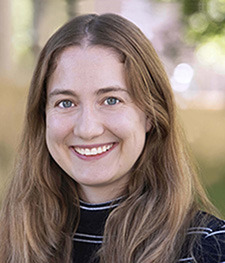


## MEET THE FIRST AUTHOR

***Meet the First Author(s)* is an editorial feature within *RNA*, in which the first author(s) of research-based papers in each issue have the opportunity to introduce themselves and their work to readers of *RNA* and the RNA research community. Aileen Button is the first author of this paper, “Dissection of protein and RNA regions required for SPEN binding to XIST A-repeat RNA.” Aileen obtained her PhD in June of 2023, working in the laboratory of Colleen McHugh at the University of California, San Diego. The McHugh lab studies noncoding RNA and protein interactions, including the XIST/SPEN interaction, which is the focus of this paper.**

**What are the major results described in your paper and how do they impact this branch of the field?**

In this paper, we used in vitro experiments to dissect the molecular determinants of the XIST/SPEN interaction. We identified a combination of RNA sequence and structure in the A-repeat of XIST that correlated with high-affinity SPEN binding. We also found that SPEN's binding to XIST uses a unique combination of residues compared to its other binding partner, SRA.

**What led you to study RNA or this aspect of RNA science?**

The McHugh lab is focused on the functional roles of lncRNAs in human development and disease. The classical example of this is XIST's ability to recruit proteins, including SPEN, resulting in the silencing of an entire X chromosome. XIST is an extreme example of lncRNAs’ ability to influence gene expression. As such, I was interested in the molecular details of this RNA/protein interaction that is so important in early female development.

**If you were able to give one piece of advice to your younger self, what would that be?**

A professor once told me that in biochemistry, there is always more pipetting to do. While this can be true, it is necessary to take time to fully understand the data rather than just continuing to do more experiments! Careful experimental planning is essential for a scientist. Deciding which experiments were important was a skill I had to learn.

**What are your subsequent near- or long-term career plans?**

I have recently started as a postdoctoral fellow in the Schwartz/Yang lab at the University of Colorado Anschutz. I am bringing my knowledge of lncRNAs to translational work on idiopathic pulmonary fibrosis.

## References

[RNA079713BUTC1] Afroz T, Cienikova Z, Cléry A, Allain FHT. 2015. One, two, three, four! How multiple RRMs read the genome sequence. Methods Enzymol 558: 235–278. 10.1016/bs.mie.2015.01.015 26068744

[RNA079713BUTC2] Aguilar R, Spencer KB, Kesner B, Rizvi NF, Badmalia MD, Mrozowich T, Mortison JD, Rivera C, Smith GF, Burchard J, 2022. Targeting *Xist* with compounds that disrupt RNA structure and X inactivation. Nature 604: 160–166. 10.1038/s41586-022-04537-z 35355011 PMC11549687

[RNA079713BUTC3] Arieti F, Gabus C, Tambalo M, Huet T, Round A, Thore S. 2014. The crystal structure of the Split End protein SHARP adds a new layer of complexity to proteins containing RNA recognition motifs. Nucleic Acids Res 42: 6742–6752. 10.1093/nar/gku277 24748666 PMC4041450

[RNA079713BUTC4] Ariyoshi M, Schwabe JW. 2003. A conserved structural motif reveals the essential transcriptional repression function of Spen proteins and their role in developmental signaling. Genes Dev 17: 1909–1920. 10.1101/gad.266203 12897056 PMC196244

[RNA079713BUTC6] Barr ML, Bertram EG. 1949. A morphological distinction between neurones of the male and female, and the behaviour of the nucleolar satellite during accelerated nucleoprotein synthesis. Nature 163: 676. 10.1038/163676a0 18120749

[RNA079713BUTC5] Basu S, Bahadur RP. 2016. A structural perspective of RNA recognition by intrinsically disordered proteins. Cell Mol Life Sci 73: 4075–4084. 10.1007/s00018-016-2283-1 27229125 PMC7079799

[RNA079713BUTC7] Brockdorff N, Ashworth A, Kay GF, McCabe VM, Norris DP, Cooper PJ, Swift S, Rastan S. 1992. The product of the mouse *Xist* gene is a 15 kb inactive X-specific transcript containing no conserved ORF and located in the nucleus. Cell 71: 515–526. 10.1016/0092-8674(92)90519-I 1423610

[RNA079713BUTC8] Brown CJ, Hendrich BD, Rupert JL, Lafrenière RG, Xing Y, Lawrence J, Willard HF. 1992. The human XIST gene: analysis of a 17 kb inactive X-specific RNA that contains conserved repeats and is highly localized within the nucleus. Cell 71: 527–542. 10.1016/0092-8674(92)90520-M 1423611

[RNA079713BUTC9] Busan S, Weeks KM. 2018. Accurate detection of chemical modifications in RNA by mutational profiling (MaP) with ShapeMapper 2. RNA 24: 143–148. 10.1261/rna.061945.117 29114018 PMC5769742

[RNA079713BUTC10] Carter AC, Xu J, Nakamoto MY, Wei Y, Zarnegar BJ, Shi Q, Broughton JP, Ransom RC, Salhotra A, Nagaraja SD, 2020. Spen links RNA-mediated endogenous retrovirus silencing and X chromosome inactivation. Elife 9: e54508. 10.7554/eLife.54508 32379046 PMC7282817

[RNA079713BUTC11] Chen F, Rebay I. 2000. *split ends*, a new component of the *Drosophila* EGF receptor pathway, regulates development of midline glial cells. Curr Biol 10: 943–946. 10.1016/S0960-9822(00)00625-4 10959845

[RNA079713BUTC12] Chu C, Zhang QC, da Rocha ST, Flynn RA, Bharadwaj M, Calabrese JM, Magnuson T, Heard E, Chang HY. 2015. Systematic discovery of Xist RNA binding proteins. Cell 161: 404–416. 10.1016/j.cell.2015.03.025 25843628 PMC4425988

[RNA079713BUTC13] Darty K, Denise A, Ponty Y. 2009. VARNA: interactive drawing and editing of the RNA secondary structure. Bioinformatics 25: 1974–1975. 10.1093/bioinformatics/btp250 19398448 PMC2712331

[RNA079713BUTC014] de Vries T, Martelly W, Campagne S, Sabath K, Sarnowski CP, Wong J, Leitner A, Jonas S, Sharma S, Allain FHT. 2022. Sequence-specific RNA recognition by an RGG motif connects U1 and U2 snRNP for spliceosome assembly. Proc Natl Acad Sci 119: e2114092119. 10.1073/pnas.2114092119 35101980 PMC8833184

[RNA079713BUTC14] Dossin F, Pinheiro I, Żylicz JJ, Roensch J, Collumbet S, Le Saux A, Chelmicki T, Attia M, Kapoor V, Zhan Y, 2020. SPEN integrates transcriptional and epigenetic control of X-inactivation. Nature 578: 455–460. 10.1038/s41586-020-1974-9 32025035 PMC7035112

[RNA079713BUTC15] Duszczyk MM, Zanier K, Sattler M. 2008. A NMR strategy to unambiguously distinguish nucleic acid hairpin and duplex conformations applied to a Xist RNA A-repeat. Nucleic Acids Res 36: 7068–7077. 10.1093/nar/gkn776 18987004 PMC2602763

[RNA079713BUTC16] Duszczyk MM, Wutz A, Rybin V, Sattler M. 2011. The Xist RNA A-repeat comprises a novel AUCG tetraloop fold and a platform for multimerization. RNA 17: 1973–1982. 10.1261/rna.2747411 21947263 PMC3198591

[RNA079713BUTC17] Elisaphenko EA, Kolesnikov NN, Shevchenko AI, Rogozin IB, Nesterova TB, Brockdorff N, Zakian SM. 2008. A dual origin of the Xist gene from a protein-coding gene and a set of transposable elements. PLoS One 3: e2521. 10.1371/journal.pone.0002521 18575625 PMC2430539

[RNA079713BUTC18] Fang R, Moss WN, Rutenberg-Schoenberg M, Simon MD. 2015. Probing Xist RNA structure in cells using targeted structure-seq. PLoS Genet 11: e1005668. 10.1371/journal.pgen.1005668 26646615 PMC4672913

[RNA079713BUTC19] Grant J, Mahadevaiah SK, Khil P, Sangrithi MN, Royo H, Duckworth J, McCarrey JR, VandeBerg JL, Renfree MB, Taylor W, 2012. *Rsx* is a metatherian RNA with *Xist*-like properties in X-chromosome inactivation. Nature 487: 254–258. 10.1038/nature11171 22722828 PMC3484893

[RNA079713BUTC20] Gruber AR, Lorenz R, Bernhart SH, Neuböck R, Hofacker IL. 2008. The Vienna RNA websuite. Nucleic Acids Res 36: W70–W74. 10.1093/nar/gkn188 18424795 PMC2447809

[RNA079713BUTC21] Hatchell EC, Colley SM, Beveridge DJ, Epis MR, Stuart LM, Giles KM, Redfern AD, Miles LE, Barker A, MacDonald LM, 2006. SLIRP, a small SRA binding protein, is a nuclear receptor corepressor. Mol Cell 22: 657–668. 10.1016/j.molcel.2006.05.024 16762838

[RNA079713BUTC22] Katoh K, Misawa K, Kuma K, Miyata T. 2002. MAFFT: a novel method for rapid multiple sequence alignment based on fast Fourier transform. Nucleic Acids Res 30: 3059–3066. 10.1093/nar/gkf436 12136088 PMC135756

[RNA079713BUTC23] Liu F, Somarowthu S, Pyle AM. 2017. Visualizing the secondary and tertiary architectural domains of lncRNA RepA. Nat Chem Biol 13: 282–289. 10.1038/nchembio.2272 28068310 PMC6432788

[RNA079713BUTC24] Lu Z, Zhang QC, Lee B, Flynn RA, Smith MA, Robinson JT, Davidovich C, Gooding AR, Goodrich KJ, Mattick JS, 2016. RNA duplex map in living cells reveals higher-order transcriptome structure. Cell 165: 1267–1279. 10.1016/j.cell.2016.04.028 27180905 PMC5029792

[RNA079713BUTC25] Lunde BM, Moore C, Varani G. 2007. RNA-binding proteins: modular design for efficient function. Nat Rev Mol Cell Biol 8: 479–490. 10.1038/nrm2178 17473849 PMC5507177

[RNA079713BUTC26] Lyon MF. 1962. Sex chromatin and gene action in the mammalian X-chromosome. Am J Hum Genet 14: 135–148. 14467629 PMC1932279

[RNA079713BUTC27] Madeira F, Pearce M, Tivey ARN, Basutkar P, Lee J, Edbali O, Madhusoodanan N, Kolesnikov A, Lopez R. 2022. Search and sequence analysis tools services from EMBL-EBI in 2022. Nucleic Acids Res 50: W276–W279. 10.1093/nar/gkac240 35412617 PMC9252731

[RNA079713BUTC28] Maenner S, Blaud M, Fouillen L, Savoye A, Marchand V, Dubois A, Sanglier-Cianférani S, Van Dorsselaer A, Clerc P, Avner P, 2010. 2-D structure of the A region of Xist RNA and its implication for PRC2 association. PLoS Biol 8: e1000276. 10.1371/journal.pbio.1000276 20052282 PMC2796953

[RNA079713BUTC29] Mathews DH, Disney MD, Childs JL, Schroeder SJ, Zuker M, Turner DH. 2004. Incorporating chemical modification constraints into a dynamic programming algorithm for prediction of RNA secondary structure. Proc Natl Acad Sci 101: 7287–7292. 10.1073/pnas.0401799101 15123812 PMC409911

[RNA079713BUTC30] Mattei E, Pietrosanto M, Ferrè F, Helmer-Citterich M. 2015. Web-Beagle: a web server for the alignment of RNA secondary structures. Nucleic Acids Res 43: W493–W497. 10.1093/nar/gkv489 25977293 PMC4489221

[RNA079713BUTC31] McHugh CA, Chen CK, Chow A, Surka CF, Tran C, McDonel P, Pandya-Jones A, Blanco M, Burghard C, Moradian A, 2015. The Xist lncRNA interacts directly with SHARP to silence transcription through HDAC3. Nature 521: 232–236. 10.1038/nature14443 25915022 PMC4516396

[RNA079713BUTC32] Minks J, Baldry SE, Yang C, Cotton AM, Brown CJ. 2013. XIST-induced silencing of flanking genes is achieved by additive action of repeat a monomers in human somatic cells. Epigenetics Chromatin 6: 23. 10.1186/1756-8935-6-23 23915978 PMC3734131

[RNA079713BUTC33] Monfort A, Di Minin G, Postlmayr A, Arieti F, Thore S, Wutz A. 2015. Identification of *Spen* as a crucial factor for *Xist* function through forward genetic screening in haploid embryonic stem cells. Cell Rep 12: 554–561. 10.1016/j.celrep.2015.06.067 26190100 PMC4530576

[RNA079713BUTC034] Montemayor EJ, Curran EC, Liao HH, Andrews KL, Treba CN, Butcher SE, Brow DA. 2014. Core structure of the U6 small nuclear ribonucleoprotein at 1.7-Å resolution. Nat Struct Mol Biol 21: 544–551. 10.1038/nsmb.2832 24837192 PMC4141773

[RNA079713BUTC0034] Moreira de Mello JC, de Araújo ES, Stabellini R, Fraga AM, de Souza JE, Sumita DR, Camargo AA, Pereira LV. 2010. Random X inactivation and extensive mosaicism in human placenta revealed by analysis of allele-specific gene expression along the X chromosome. PLoS One 5: e10947. 10.1371/journal.pone.0010947 20532033 PMC2881032

[RNA079713BUTC34] Penny GD, Kay GF, Sheardown SA, Rastan S, Brockdorff N. 1996. Requirement for *Xist* in X chromosome inactivation. Nature 379: 131–137. 10.1038/379131a0 8538762

[RNA079713BUTC35] Sahakyan A, Yang Y, Plath K. 2018. The role of *Xist* in X-chromosome dosage compensation. Trends Cell Biol 28: 999–1013. 10.1016/j.tcb.2018.05.005 29910081 PMC6249047

[RNA079713BUTC36] Shi Y, Downes M, Xie W, Kao HY, Ordentlich P, Tsai CC, Hon M, Evans RM. 2001. Sharp, an inducible cofactor that integrates nuclear receptor repression and activation. Genes Dev 15: 1140–1151. 10.1101/gad.871201 11331609 PMC312688

[RNA079713BUTC37] Smola MJ, Weeks KM. 2018. In-cell RNA structure probing with SHAPE-MaP. Nat Protoc 13: 1181–1195. 10.1038/nprot.2018.010 29725122 PMC6402486

[RNA079713BUTC38] Sun L, Fazal FM, Li P, Broughton JP, Lee B, Tang L, Huang W, Kool ET, Chang HY, Zhang QC. 2019. RNA structure maps across mammalian cellular compartments. Nat Struct Mol Biol 26: 322–330. 10.1038/s41594-019-0200-7 30886404 PMC6640855

[RNA079713BUTC39] Wiellette EL, Harding KW, Mace KA, Ronshaugen MR, Wang FY, McGinnis W. 1999. *spen* encodes an RNP motif protein that interacts with Hox pathways to repress the development of head-like sclerites in the *Drosophila* trunk. Development 126: 5373–5385. 10.1242/dev.126.23.5373 10556062

[RNA079713BUTC40] Wutz A, Rasmussen TP, Jaenisch R. 2002. Chromosomal silencing and localization are mediated by different domains of *Xist* RNA. Nat Genet 30: 167–174. 10.1038/ng820 11780141

